# Climate Change Effects on Grapevine Physiology and Biochemistry: Benefits and Challenges of High Altitude as an Adaptation Strategy

**DOI:** 10.3389/fpls.2022.835425

**Published:** 2022-05-26

**Authors:** Leonardo A. Arias, Federico Berli, Ariel Fontana, Rubén Bottini, Patricia Piccoli

**Affiliations:** ^1^Grupo de Bioquímica Vegetal, Instituto de Biología Agrícola de Mendoza, CONICET, Chacras de Coria, Argentina; ^2^Instituto Argentino de Veterinaria, Ambiente y Salud, Universidad Juan Agustín Maza, Guaymallén, Argentina

**Keywords:** environmental stress, global warming, secondary metabolites, *Vitis vinifera*, temperature, UV-B radiation, viticulture

## Abstract

Grapevine berry quality for winemaking depends on complex and dynamic relationships between the plant and the environment. Winemakers around the world are demanding a better understanding of the factors that influence berry growth and development. In the last decades, an increment in air temperature, CO_2_ concentration and dryness occurred in wine-producing regions, affecting the physiology and the biochemistry of grapevines, and by consequence the berry quality. The scientific community mostly agrees in a further raise as a result of climate change during the rest of the century. As a consequence, areas most suitable for viticulture are likely to shift into higher altitudes where mean temperatures are suitable for grape cultivation. High altitude can be defined as the minimum altitude at which the grapevine growth and development are differentially affected. At these high altitudes, the environments are characterized by high thermal amplitudes and great solar radiations, especially ultraviolet-B (UV-B). This review summarizes the environmental contribution of global high altitude-related climatic variables to the grapevine physiology and wine composition, for a better evaluation of the possible establishment of vineyards at high altitude in climate change scenarios.

## Introduction

Grapevine cultivation for winemaking is highly dependent on climate. Normally, it requires mean air temperatures ranging from 12°C to 22°C during the growing cycle, at least 700–900 μmol photons m^−2^ s^−1^ solar radiation, around 600–800 mm rainfall or sufficient irrigation, and a frost-free growing season (Palliotti and Poni, 2016; Zapata et al., 2016; Ateş and Uysal, 2017). This generally restrains most grape growing beyond 50° latitude N and 40° latitude S (Kenny and Shao, 1992; Cabré et al., 2016).

High-altitude viticulture has been acquiring great importance in the last decades due to its potential to produce high-quality wines in warmer regions, whether by nature of its geography or by an advancement of climate change. Certain countries, namely Argentina, Brazil, China, Italy, Portugal and Turkey, have taken part in an expansion both in high-altitude grape cultivation and in its scientific study. Table 1 summarizes the current literature regarding vineyard altitude and its effect on several grapevine and wine characteristics. Particularly, these countries cultivate at higher altitudes than the average for wine growing regions (*ca*. 300 m a.s.l.; Gladstones, 2011). What is considered high-altitude vineyard in the current literature ranges from 350 m a.s.l. in Douro Valley (Oliveira et al., 2004) to 2,900 m a.s.l. in southwest China (Xing et al., 2016). This allows us to define high altitude in relative terms, pointing out the minimum altitude change that differentially affects grapevine growth and development, as well as wine quality, for a given region.

**Table 1:** Research papers studying different magnitudes of high altitude and their effect in grapevine.

Altitude	Region	Variables	Measured effects	References
950–1,350	Argentina (Mendoza)	Altitude, Site	Yield, Phenolic compounds	Muñoz et al., 2021
635–1,500	Argentina (Mendoza)	Altitude, Site	Phenolic compounds	Urvieta et al., 2021
635–1,500	Argentina (Mendoza)	Altitude, Site	Phenolic compounds, Sensory profiles	Urvieta et al., 2018
1,500	Argentina (Mendoza)	UV exclusion	Berry biochemistry, Antoxidant capacity	Berli et al., 2015
1,500	Argentina (Mendoza)	UV exclusion	Volatile organic compounds	Gil et al., 2013
1,500	Argentina (Mendoza)	UV exclusion	Growth, Gas exchange, Antioxidant capacity	Berli et al., 2015
500, 1,000, 1,500	Argentina (Mendoza)	Altitude, UV exclusion	Phenolic composition	Berli et al., 2008
1,100	Brazil (Minas Gerais)	Grafting combinations	Physiology, biochemistry	Souza et al., 2015
873, 1,150	Brazil (Minas Gerais)	Altitude	Phenology, biochemistry	Regina et al., 2010
1,100	Brazil (Northeast)	Cultivar	Oenological potential	Torres et al., 2013
774–1,415	Brazil (Santa Catarina)	Altitude, Temperature	Organoleptic properties	Falcão et al., 2007
675–1,452	Brazil (Santa Catarina)	Altitude, Cultivar	Edaphoclimatic characteristics	Vianna et al., 2016
1,230	Brazil (Santa Catarina)	Leaf removal	Yield, Phenology, Biochemistry	Würz et al., 2017
1,400	Brazil (Santa Catarina)	Cultivar	Phenology, Biochemistry	Brighenti et al., 2014
950, 960	Brazil (Santa Catarina)	Site	Phenology, Phenolic maturation	Malinovski et al., 2010
1,350	Brazil (Santa Catarina)	Season, training	Leaf parameters	Malinovski et al., 2013
1,290	Brazil (Santa Catarina)	Cultivar, Vintage	Proanthocyanidin profile	Gris et al., 2011a
1,230	Brazil (Santa Catarina)	Cluster thinning	Polyphenols	Silva et al., 2009
1,290	Brazil (Santa Catarina)	Cultivar, Vintage	Antioxidant capacity, Hypolipidemic activity	Gris et al., 2011b
774–1,415	Brazil (Santa Catarina)	Altitude, Temperature	Phenology, Grape maturation	Falcão et al., 2010
950, 1,400	Brazil (Santa Catarina)	Altitude	Phenology, Thermal requirements	Muniz et al., 2015
1,290	Brazil (Santa Catarina)	Altitude	Chemical composition	Gris et al., 2010
46–255	Chile	Site	Phenolic composition	Martínez-Gil et al., 2018
2,150, 2,300, 2,900	China	Altitude, Vintage	Flavonoids	Xing et al., 2016
2,110–2,788	China	Altitude	Aroma (GC–MS)	Yue et al., 2015
1900–3,500	China	Altitude, Site	2dary Metabolism	Li et al., 2011
50–2,500	China	Cultivar	Anthocyanin profile	He et al., 2010
2,282, 2,435, 2,608	China	Altitude	Antioxidant capacity, Sensory evaluation	Jin et al., 2017
212–1,214	China	Site, Cultivar	Anthocyanin, Flavonols	Liang et al., 2014
270–730	Greece	Altitude, Site	Biochemistry	Petropoulos et al., 2017
300,500,700	Greece (Southern)	Altitude, Irrigation, Direction	Phenology, Biochemistry, Organoleptic	Koundouras et al., 2006
100–550	Italy	Altitude, Site	Phenology	Caffarra and Eccel, 2010
131–600	Italy (Trentino)	Altitude, Temperature	Bud burst, Flowering, Veraison	Caffarra and Eccel, 2011
210–650	Italy (Trento)	Altitude, Temperature	Bud burst, Harvest, TGS duration	Alikadic et al., 2019
50, 280, 595	Macedonia	Altitude, Pruning	Grape Anatomy	Nedelkovski et al., 2018
25–400	Montenegro	Altitude, Site	Polyphenols	Pajovic et al., 2014
85–210	Portugal (Douro)	Altitude, Cultivar., Sunlight	Carotenoid profile	Oliveira et al., 2004
100–350	Portugal (Douro)	Altitude	Phenology, Biochemistry	Mateus et al., 2001a
100–350	Portugal (Douro)	Altitude	Polyphenolic composition of grape and wine	Mateus et al., 2001b
100–350	Portugal (Douro)	Altitude, Cultivar	Polyphenolic composition	Mateus et al., 2002
148–413	South Africa	Altitude	Phenology, Grape composition	Conradie et al., 2002
100–1,000	Spain	Altitude, Site	Adaptation efforts	Resco et al., 2016
527	Spain (La Rioja)	UV exclusion	Physiology, 2dary Metabolism	Del-Castillo-Alonso et al., 2015
371	Spain (Navarra)	UV exclusion	Physiology, 2dary Metabolism	Del-Castillo-Alonso et al., 2016
280–520	Spain (Tenerife)	Altitude	Biochemistry	Miguel-Tabares et al., 2002
180, 280, 405	Turkey	Altitude, Direction	Leaf Area; Stomatal characteristics	Kok and Bahar, 2015
1,000, 1,500	Turkey	Altitude	Antioxidant capacity, 2dary Metabolism	Coklar, 2017
800–1,150	Turkey	Altitude	Genetic variation	Agar et al., 2012a
800, 900, 1,000, 1,150	Turkey	Altitude	Genetic, Biochemistry	Agar et al., 2012b
195	Turkey	Site	Suitability for viticulture	Köse, 2014
10–1,223	Turkey	Altitude, Site	Adaptation level	Ateş and Uysal, 2017

According to Körner (2007), environmental variables that change with altitude can be grouped into two categories: those derived from altitude *per se*, such as temperature, atmospheric pressure and sky turbidity; and those tied to proximity to hilly and mountainous terrain, such as moisture, wind and geology. This review focalizes in the former, by virtue of being globally comparable, while the latter are too site specific to summarize.

## Climate Change: What Is Changing?

According to the sixth assessment report of the Intergovernmental Panel on Climate Change (IPCC), the last four decades have been sequentially warmer than any other decade since 1850 (IPCC, 2021). Particularly, land surface temperature in the first two decades of the twenty-first century was 1.59°C higher than 1850–1900. Hot extremes have become more frequent and more intense, while cold extremes have become less frequent and severe since 1950. Additionally, since 1750, CO_2_ concentrations have continually increased due to human activities, reaching annual averages of 410 ppm. Alarmingly, it is very likely that most of these aspects will persist for many centuries even when CO_2_ emissions were to be stopped (Gillett et al., 2011; IPCC, 2013).

The IPCC has carried out several projections of climate change based on a set of scenarios of anthropogenic impact, from more optimistic to more pessimistic, which would result in different magnitude of emissions, or Representative Concentration Pathways (RCPs). These include an increase in global mean surface temperatures of 1.7°C (RCP2.6) to 4.8°C (RCP8.5) for the period 2081–2100 relative to 1986–2005, an increase in atmospheric CO_2_ concentration reaching 421 ppm (RCP2.6) to 936 ppm (RCP8.5) by the year 2100, and a virtual certainty of more frequent hot and fewer cold temperature extremes (IPCC, 2013).

Given this scenario, major impacts are to be expected in rural systems, including shifts in production areas of food crops, as well as adaptation strategies such as more adequate cultural practices and crop varieties (IPCC, 2014; Fraga, 2020).

## CC and Grapevine Phenology

Webb et al. (2012) carried out a modelling study of temperature and non-temperature climatic drivers of wine-grape maturity in southern Australia. The harvest date was consistently best represented by the average temperatures during the growing season across sites, followed by lower soil moisture. Shifts of between 4 and 11 days were attributed to warming. Reduced soil moisture advanced maturity independently of increasing temperature, although recent drying trends in Australia have been attributed to anthropogenic climate change.

Arrizabalaga-Arriazu et al. (2020b) determined the phenological response of Tempranillo clones to the air temperature and CO_2_ concentration predicted by 2100. In general, among clones, elevated temperature reduced the total time needed to reach fruit maturity. CO_2_ did not have significant effects on plant phenology, with the exception of RJ43 clone, which was the most affected by climate change scenarios. This revealed the importance of plant material and genetic variability for climatic adaptation.

Ruml et al. (2016) examined the historical onset dates of budburst, flowering, veraison and harvest for 20 wine grape cultivars for the period 1981–2007. Although all phenological stages advanced significantly, the greatest thermal effect was observed from the beginning of flowering to the beginning of veraison, while the smallest occurred during the ripening period. Trends were − 0.4 day/year for flowering, −0.7 days/year for beginning of veraison, and −0.6 day/year for harvest date. Authors also found nonlinear effects of warming, explaining up to 26% of the variation in phenology timing, attributed to a shift of the ripening period into warmer conditions earlier in the season due to precocious flowering and veraison. Surprisingly, a 1°C increase in average temperature during the beginning of budburst, flowering or veraison was enough to advance average harvest times by 7.4 days.

In greenhouse experiment settings simulating temperature, CO_2_ and water deficit conditions expected for the end of the century, Martínez-Lüscher et al. (2016a,b) showed a significant advancement of budburst, flowering and berry maturity in Red and White Tempranillo. Conditions with 700 ppm CO_2_ and 28°C resulted in an earlier onset of veraison of about 9 days, and harvest time (22 °brix) by around 23 days. Overall, the effect of temperature on maturity was between −0.90day/°C and − 2.03 days/°C across years and varieties, while the effect of CO_2_ for all years and varieties was an advancement of phenology times by 6.75 days for ambient temperature and 4.06 days for T + 4°C.

Hall et al. (2016) modelled budbreak and harvest events with temperature data interpolated from 1975 to 2004, and projected warmer conditions across different emissions scenarios in Australian winegrowing regions. This study suggests that budbreak and fruit maturity will happen sooner in all Australian regions, consequently increasing post-harvest heat accumulation specially for cooler regions. This would cause an accumulation of carbohydrates and nutrient reserves in the perennial structures, likely enhancing vigor and vegetative growth in the following year.

Ramos (2017) made phenology predictions of three grape varieties Chardonnay, Macabeo and Parellada for a Mediterranean climate (Spain) with no irrigation for the years 2030, 2050 and 2070 according to RCP4.5 and RCP8.5. The study suggested an earlier onset of all phenological stages, mainly veraison and harvest, with a shortening of both the phenological timing and the intervals between phases, which may affect grape quality. This was also true for Tempranillo variety (Ramos and de Toda, 2020). Additionally, a reduction of available water due to higher evapotranspiration may accentuate the advance of some phenological stages, namely veraison. Veraison and harvest could advance up to 12 and 20 days for 2070 under RCP4.5 scenario, and 23 and 28 days under RCP8.5 scenario.

## CC and Grapevine Physiology

Schultz and Hofmann (2016) reviewed how rising CO_2_ to about 560 ppm will cause an overall decrease in air relative humidity and an increased evaporative demand by means of reduced stomatal conductance of about 20%. With double CO_2_, stomatal conductance would be reduced by around 40%, in turn reducing transpiration by 20%, evapotranspiration by 10%–14%, and less evaporative cooling should result in a higher temperature experienced by plant surfaces. In areas without irrigation, such levels of deficit would not be desirable in terms of wine quality. Apart from the indirect effect of CO_2_ on global temperature, CO_2_ itself should be beneficial to grapevine growth, increasing biomass production at a reduced water loss, and ultimately increasing fruit sugar concentration and decreasing acidity levels.

Salazar-Parra et al. (2012) assessed the influence of CC conditions (elevated temperature and CO_2_, and moderate water deficit) on the antioxidant condition of Tempranillo leaves from veraison to maturity. Elevated ambient temperature (28°C) and CO_2_ (700 ppm) presented lower membrane lipid peroxidation and ROS concentrations (as H_2_O_2_) in leaves, particularly under well irrigated conditions as compared to water deficit. It was attributed to increases in carbon fixation from photosynthesis. Leaves under moderate water stress and present ambient conditions (375 ppm and 24°C) were more susceptible to oxidative damage, indicating that the antioxidant system was less able to alleviate the damage (Salazar-Parra et al., 2012).

Arrizabalaga-Arriazu et al. (2020b) studied the physiological response of five clones of Tempranillo to the temperature and CO_2_ predicted by the year 2100 in a greenhouse experiment. Net photosynthesis was initially stimulated at veraison, but down regulation of photosynthesis occurred because of elevated CO_2_ combined with high temperature causing nitrogen limitation. It has been largely known that CO_2_ concentration negatively correlates with stomata conductance (Lammertsma et al., 2011).

## CC and Grape Biochemistry

Martínez-Lüscher et al. (2016b) found that elevated CO_2_ and increased temperature lowered the anthocyanin to sugar ratio in grape berries, along with earlier onset of veraison and higher total soluble solids.

Van Leeuwen and Destrac-Irvine (2017) focused on the effect of temperature, UV-B and water status in viticulture. The combined effect of increased temperatures and advanced phenology caused more sugar and less organic acids in berries, and altered secondary metabolites composition, particularly aroma precursors.

In a field study aimed to assess the effect of +20% supplemented CO_2_ in Riesling and Cabernet Sauvignon wines, Wohlfahrt et al. (2021) reported no or little effect of CO_2_ in organic acids and sugars in grape must, but in the ratio of tartaric to malic acid, tartaric acid was favored for both cultivars. There was, however, a lower anthocyanin concentration due to the indirect effect CO_2_ increasing berry size. Principal component analyses revealed that the vintage effect was overall stronger than the +20% elevated CO_2_ treatment (Wohlfahrt et al., 2021). Similarly, Arrizabalaga-Arriazu et al. (2020a) studied must composition and skin total anthocyanins for Tempranillo clones under the climatic conditions predicted for 2,100. Increases in CO_2_ and average air temperature are predicted to reduce time to ripening and enhance sugar accumulation and malic acid breakdown, while delaying amino acidic maturity. Even though anthocyanin concentration is not predicted to change under these conditions, there could be a reduction of the ratio between anthocyanins and sugars. The degree to which high temperature affects the anthocyanin to sugar ratio is believed to be cultivar dependant, due to different sensitivity of berry anthocyanin to critical ranges of temperature (Fernandes de Oliveira et al., 2015). Furthermore, Ramos and de Toda (2020) projected both optimistic and pessimistic CC scenarios for grape composition, and estimated decreasing values of total acidity in three analyzed zones from the projected changes in temperature.

## Shifts in Viticultural Distribution

The magnitude to which environmental factors constraint viticulture for grape production differ considerably from region to region (Schultz and Hofmann, 2016). Likewise, the impacts of climate change will vary accordingly. Sites where viticulture is characterized by short growing seasons or low summer temperatures, like those located in higher latitudes or elevation, are expected to get progressively warmer under future climate conditions, allowing for a wider selection of cultivars to be reliably grown (Mosedale et al., 2016). Conversely, current premium grape producing regions may suffer major reductions in quality due to projected increase in temperature and lower water availability (Mosedale et al., 2016).

The Huglin Index (HI) has been shown to be an effective tool for viticultural zoning and has been thus widely applied (Jones et al., 2010). Fraga et al. (2013) analyzed the simulated HI patterns for the period 2041–2070 in Europe and showed a northward displacement of high suitability areas for viticulture. Southern European regions, on the other hand, will have reduced suitability mainly due to increased dryness, and large areas of central and western Europe will become more suitable due to favorable thermal conditions (Fraga et al., 2013).

Mosedale et al. (2015) suggested that under future climate scenarios, even under low emissions, there will be a much wider range of cultivars suitable for high quality harvest of wine grapes in cool climate regions. South-East England, for example, currently is adequate to produce high-quality sparkling wine, which require particularly acidic grapes, but in future scenarios it could permit reliable harvests of Chardonnay or Pinot Noir (Mosedale et al., 2015).

Lorenzo et al. (2016) evaluated four different regional climate models (RCMs) for Spain, using three bioclimatic indices for winegrowing (Huglin and Branas, Winkler, and Bernon and Levadoux). All RCMs agree on an increased accumulation of heat in central and southern Spain, which would have a negative impact on wine quality (Lorenzo et al., 2016). It is further projected that by the end of the twenty-first century, southern Spain would be no longer suitable for high-quality wine production due to the thermal hindrance on grapevine growth (Lorenzo et al., 2016). On the other hand, Northwestern Spain could benefit from projected warming, increasing potential wine quality (Lorenzo et al., 2016). It is noted that different bioclimatic indices disagree on the size of future projected change.

Cabré et al. (2016) also analyzed a regional climate model simulation through several bioclimatic indices, for the Cuyo region in Argentina, where most of the country’s wines are produced. The projections predict a displacement of suitable areas southward, to higher latitudes, and westward, to higher latitudes, to accommodate for the change in temperature by the end of the century (2075–2099; Cabré et al., 2016; Cabré and Nuñez, 2020). Thus, under projected scenarios, current Argentina’s main wine producing region would face great adaptation challenges for the development of viticulture, while southern provinces and higher regions would be benefited by favorable growing conditions (Cabré and Nuñez, 2020).

Teslić et al. (2019) stated that for the period 2011–2040, the Italian region Emilia-Romagna will still be suitable for grape production under RCP4.5 and RCP8.5 scenarios, but the period 2071–2100 will likely be too hot to maintain current yield and quality of grapes.

Using a similar approach, Cardell et al. (2019) found that the general increase projected for maximum temperatures across Europe could lead to the loss of viability for winemaking of some regions in the near future if no adaptive measures were implemented. This is mainly due to the plant being exposed to temperatures that exceed the maturation threshold for longer periods (Cardell et al., 2019). However, countries that currently have a colder ground temperature, such as Germany, North France, Belgium, South England and Czech Republic, would reach more favorable thermal conditions by the mid-century (Cardell et al., 2019).

Schultz (2016) has argued that the underlying assumptions concerning the suitability of certain sets of climate, region and varieties for grapevine cultivation are decisive, and have been based partly on the misuse of temperature summation index, such as HI. Although there is a clearer lower limit of growing temperature for each variety, the upper limit has been progressively pushed forward in many regions without negative effects on wine quality (Schultz, 2016).

Naulleau et al. (2021) noticed that local conditions, such as soil available water capacity and irrigation water availability are typically not integrated into suitability mapping studies. This, together with land use conflicts arising from conservation policies and the preference for other crops, could turn out to be major constraints for establishing new wine growing territories (Naulleau et al., 2021). Another aspect that is not considered is the plant’s ability to acclimate to different environmental conditions, which in turn may be epigenetically inheritable (Marfil et al., 2019).

As an example, the warming expected for other colder regions by the end of the century, such as the case of Scotland, still will not make wine grape production plausible due to a concomitant increase in precipitations in already wet areas (Dunn et al., 2019).

## Altitude as an Alternative

Skarbø and VanderMolen (2016) noted that over the past two decades, farmers in Northern Ecuadorian highlands have expanded cultivated area about 200–300 m in elevation as an adaptive response to recent changes in growing conditions driven by global warming.

Judging by the literature of the last decade, higher altitude regions have become a recognized alternative for maintaining current high-quality winegrowing of traditional varieties in future climate scenarios, mostly due to a lower mean air temperature (Cabré et al., 2016; Lorenzo et al., 2016; Van Leeuwen and Destrac-Irvine, 2017; Gaiotti et al., 2018; Ramos and de Toda, 2020; Naulleau et al., 2021; Mansour et al., 2022). It is, nevertheless, an adaptation strategy only possible in certain regions.

What has not been pointed out, is the new challenges that higher altitudes may pose to future viticulture, associated with the environmental factors that change with elevation.

**Figure 1:**
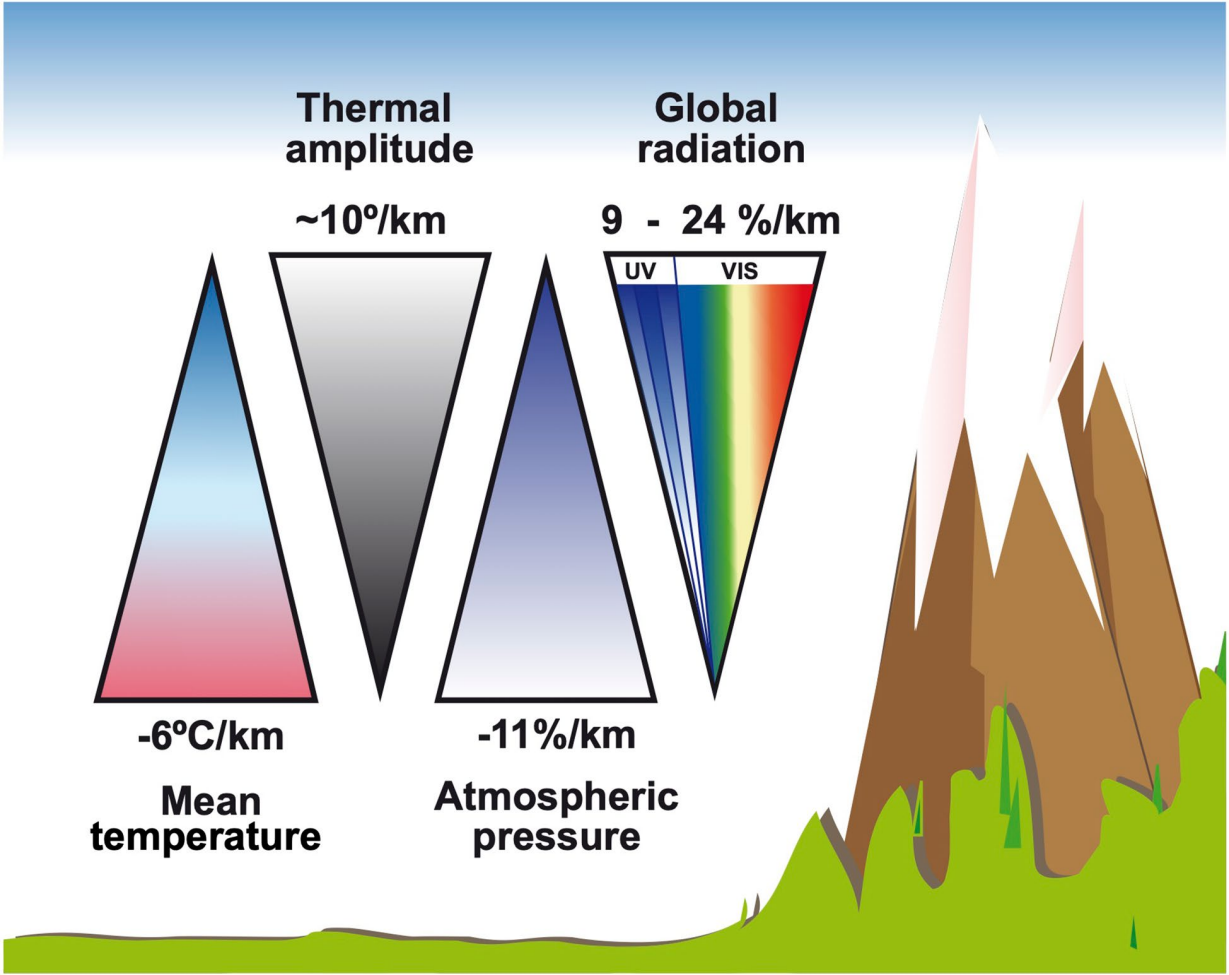
Global climatic parameters as they vary with increasing altitude.

The primary weather changes associated with an altitude raise are the decrease of air temperature, the increase of thermal amplitude and a higher net global radiation exchange (that is, incoming solar radiation and outgoing thermal radiation), accompanied with a higher proportion of UV-B radiation (Körner, 2007). For example, the maximum irradiance value of solar UV-B registered during a summer day in Mendoza, Argentina (32°32′S, 69°W) increased by 15% from 1,231 m a.s.l. to 2,393 m a.s.l. (32.5 and 37.4 μW cm^−2^, respectively), while photosynthetically active radiation (PAR) increased 2.6% for the same comparisons (Ibañez et al., 2017). This has been found to be dependant on the solar elevation angle along the day, which can affect irradiance non-linearly in certain regions (Piazena and Häder, 2009). Figure 1 illustrates how global climatic parameters change with the altitudinal gradient.

## Altitude and Phenology

Phenology is the study of periodic events (stages of growth and development) in biological life cycles and how these are influenced by the environment. Such information is necessary for decisions on site, cultivar selection and cultural practices (Keller, 2020). The major phenological stages for *Vitis vinifera* L. are: budbreak, flowering, fruit-set, pea-size berries, veraison (onset of ripening) and harvest (Coombe, 1995). Phenological stages are highly influenced by temperature, thermal amplitude and solar radiation, which together determine the length of phenophases for a given variety (Zapata et al., 2016).

Falcão et al. (2010) evaluated the influence of vineyard altitude (from 774 to 1,415 m a.s.l.) on the phenology and maturation of Cabernet Sauvignon grapes in Santa Catarina, Brazil (latitudes 26°–28° S). They reported up to 4°C lower mean air temperatures at higher altitudes, which delayed harvest time, measured as berry total soluble solids concentration (°Brix), up to 2 months as compared to lower altitudes. Muniz et al. (2015) observed the same trend in high altitude regions of southern Brazil. The vineyard at the highest altitude (1,400 m a.s.l.) had a mean air temperature up to 4.4°C lower, and received 20% more PAR and 12% more total radiation at the daily maximum as compared to a vineyard at 950 m a.s.l. This variation in the cultivation site dramatically affected the length of phenophases, with a delay of 50–56 days in harvest dates and 37 days from budbreak to full bloom of both Merlot and Cabernet Sauvignon cultivars.

In a terroir study in Switzerland, Rienth et al. (2020) analyzed how pedoclimatic conditions interact with grapevine phenology. While there were differences in budburst and flowering driven mainly by altitude (375–575 m a.s.l.), the vintage climatic variations in the 3-year study did not yield consistent results.

Gonzalez Antivilo et al. (2017) demonstrated marked differences in the thermal amplitudes registered between two contrasting regions of America (Mendoza, Argentina and Prosser, Washington State, United States). In Mendoza, both the average and the minimum temperatures are higher than in Prosser, reducing the risk of frost damage during winter. Nevertheless, the considerably higher thermal amplitude at 766 m a.sl. in Mendoza (up to 10° higher than Prosser at 117 m a.sl.) means that there will be unusually hot days during winter, which can cause cold deacclimation and subsequent cold injury in late winter (Gonzalez Antivilo et al., 2017).

Gaiotti et al. (2018) evaluated the effect of lower night temperatures on Corvina grape berry quality. Potted plants were transferred to a cold room overnight starting before the onset of coloration and continued until full veraison, which delayed veraison by about 6 days.

The use of phenological models to predict the length of phenophases from temperature and global radiation information has become increasingly frequent (Ramos et al., 2018; Alikadic et al., 2019; Hall and Blackman, 2019; Leolini et al., 2020). Alikadic et al. (2019) described the grapevine development in the winemaking region of Trento, Italy, characterized by marked altitudinal differences, utilizing the FENOVITIS model (Caffarra and Eccel, 2010). They studied five varieties (Pinot Noir, Sauvignon Blanc, Chardonnay, Merlot, Pinot Gris) and assessed statistical correlation between altitude (67–950 m a.s.l.) and the timing of budbreak, harvest and the length of budbreak to harvest interval. The harvest time was remarkably influenced by altitude and varieties, ranging from 6.27 to 7.16 days every 100 m of elevation, while budbreak time ranged from 0.85 to 2.88 days every 100 m.

**Figure 2:**
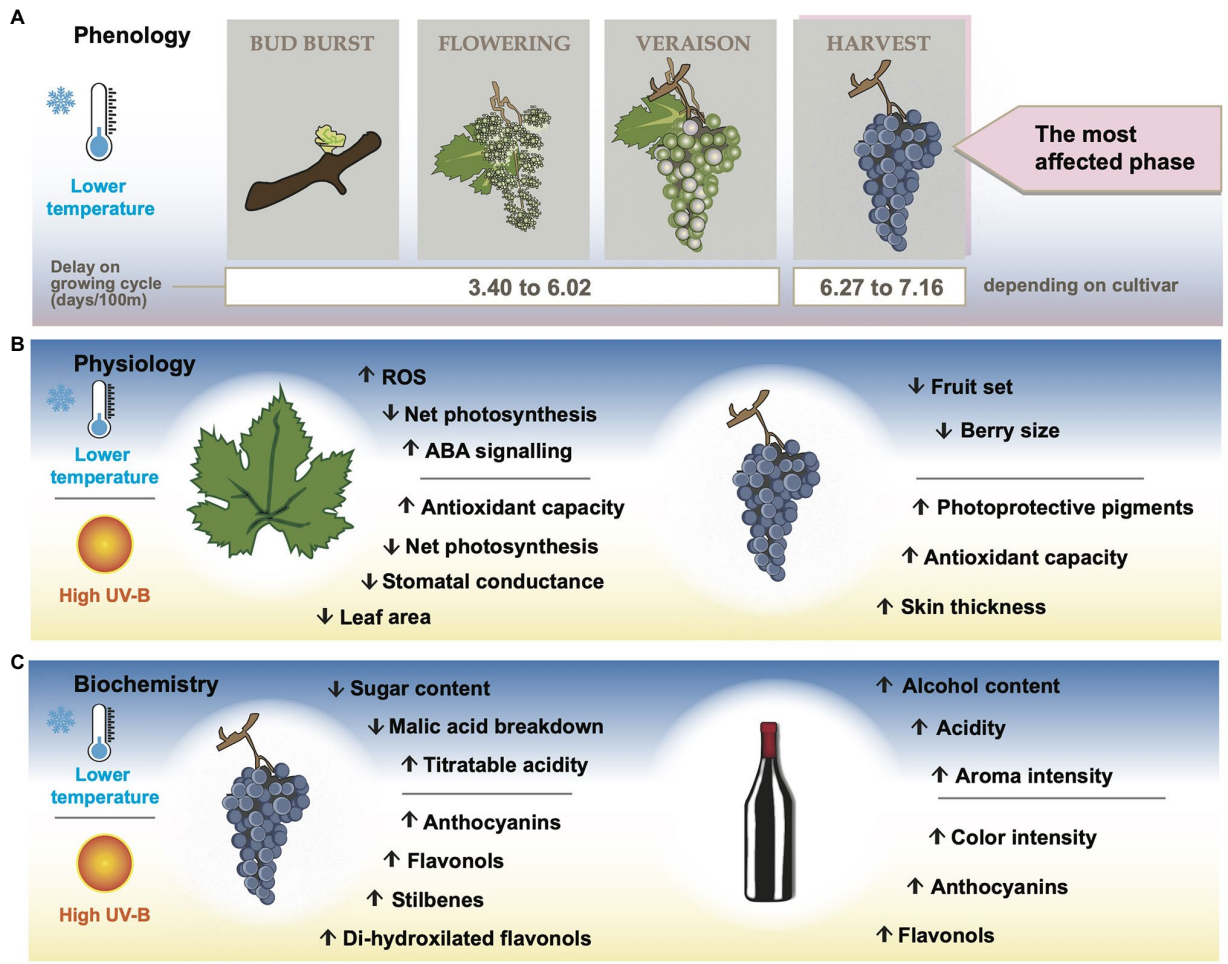
Effects of low temperature and high UV-B at higher altitude vineyards on grapevine phenology **(A)**, physiology **(B)**, grape biochemistry, and wine chemical composition **(C)**.

It can thus be proposed that, on a global scale, an increase in altitude causes a delay of phenological phases (Figure 2A). The major effects are seen in harvest times followed by budbreak, which might increase the risk of hail damage and frost, respectively, by prolonging the period of vulnerable structures exposed to the cooler part of the growing season. On the other hand, longer phenological phases may allow that certain cultivars with shorter growing cycles reach superior oenological levels by helping synchronize grape maturation with sufficient accumulation of aromatic and colored compounds.

## Altitude and Physiology

Key physiological processes affected by altitudinal-related environmental conditions take place during shoot elongation and berry development stages (Pillet et al., 2015; Palliotti and Poni, 2016; Sawicki et al., 2016). Grapevine physiology can be affected (even damaging the plant), by stressful or limiting factors. They may include excessive temperatures in warm climates (especially at low altitude), low temperatures in temperate/cool climates and/or high altitudes, and extreme solar UV-B radiation at very high altitudes (Körner, 2007; Figure 2B).

High temperature may hamper grapevine vegetative growth and reproductive development, impacting fruit yield and quality. Photosynthesis is one of the most heat-sensitive processes, often being impeded before other stress symptoms become apparent. In general, temperatures above 35°C reduce photosynthesis in leaves by the combined effect of restrained stomatal conductance (Greer and Weedon, 2012), impaired photosystem II reaction center and altered activation state of Rubisco (Luo et al., 2011). Conversely, higher altitudes can carry higher occurrence of cold days and frost risk that come along not only with generally lower temperatures but also with higher thermal amplitude (Körner, 2007). One-year grapevine shoots are particularly sensitive to injury at temperatures below −2.5°C, causing the loss of primary buds or flowers (Sawicki et al., 2016). Rooy et al. (2017) evaluated the effects of chilling by gradually decreasing the air temperature for three table grape cultivars to 0°C and −4°C. They found a significant increase in cell electrolyte leakage and oxidative stress biomarkers (H_2_O_2_ and thiobarbituric acid) in leaves, purportedly as a result of lipid peroxidation and membranes injury (Rooy et al., 2017). Furthermore, chilling can reduce stomatal conductance and net photosynthesis (Sawicki et al., 2012; Król et al., 2015; Rooy et al., 2017), and even affect the fruit set and carbon accumulation in berries (Sawicki et al., 2016).

An increase in the concentration of leaf proline and ABA has been also observed during the cold tolerance response on three grapevine cultivars (Rooy et al., 2017). This ABA pathway activation can indirectly lead to a sounder abiotic stress tolerance due to an enhancement of the plant antioxidant system (Berli and Bottini, 2013; Carvalho et al., 2015). Alonso et al. (2015) observed this effect by spraying ABA on leaves and berries from veraison onwards, which promoted the accumulation of antioxidant and antifungal compounds. Cooler temperatures in higher altitudes can also extend berry maturation periods, which favors a positive net turnover of organic acids, higher biosynthesis of flavonols and anthocyanins, resulting in a more suitable fruit for red winemaking (Muniz et al., 2015; Martínez-Gil et al., 2018).

In plants, light acts both as source of energy for photosynthesis and as an environmental signal eliciting photomorphogenic responses. In higher altitudes and during the grapevine growth period, the solar PAR is mostly over the 700–900 μmol photons m^−2^ s^−1^, which is the saturation point for grapevine. Leaves exposed to excess of light usually display a series of preventive mechanisms to avoid photoinhibition and oxidative damage. In order to diminish the light absorbance, leaves can reduce its blade area, adjust its chlorophyll concentration and accumulate photoprotective pigments, such as carotenoids and flavonoids including anthocyanins (Palliotti and Poni, 2016). Kok and Bahar (2015) described a 42% decrease in leaf area, along with a 34% increase in stomatal density, in Gamay cultivar from 405 m a.s.l. as compared to those cultivated at lower altitudes (180 m a.s.l.). Grape berry skin was also found to respond to excess radiation through morphochemical changes. A difference in altitude from 50 to 595 m a.s.l. caused 28% thicker hypodermal layer in the skin of Vranec berries (Nedelkovski et al., 2018). The hypodermis of red varieties accumulates anthocyanins, which serve as photoprotective pigments during berry development (Cadot et al., 2011; de Alencar Filho et al., 2016); and their accumulation is promoted as elevation increases (Berli et al., 2008).

The high-altitude and most reputed vineyards in Mendoza, Argentina, are located in Gualtallary at *ca*. 1500 m a.s.l. (33°23′S, 69°15′W), with UV-B irradiances that reach up to 40 μW cm^−2^ in summer days, while at sea level it is measured around 15 μW cm^−2^ (Berli et al., 2010). The defense system of grapevine leaves against UV-B include the accumulation of photoprotective phenolic pigments, and the activation of enzymatic and non-enzymatic antioxidative mechanism, with the phytohormone abscisic acid (ABA) acting downstream in the signaling pathway (Berli et al., 2010). The effects of UV-B on grapevine’s physiology was evaluated through in field experiments with solar UV-B exclusion by plastic sheeting (from flowering to harvest) during different growing seasons (Berli et al., 2010). Berli et al. (2013) found that the effects of UV-B on vegetative growth are variable and interplay with other seasonal environmental conditions such as air temperature. Generally, high-altitude solar UV-B reduced shoot length, leaf expansion, photosynthesis and stomatal conductance; and augmented leaf thickness, photoprotective pigments, proline accumulation and the antioxidant capacity of leaves (Berli et al., 2013; Martínez-Lüscher et al., 2013). In addition, high UV-B reduced berry size and fruit yield and improved grape berry skin phenolic compounds (Berli et al., 2011) and antioxidant capacity (Berli et al., 2015).

In a similar experiment with the Graciano variety, Del-Castillo-Alonso et al. (2015) did not find differences between leaves submitted to UV-B exclusion and the UV-B exposed controls. This lack of response may be attributed to an insufficient UV-B differential, short exclusion period (in this experiment the treatment started at veraison), sampling of old leaves (less sensitive to UV-B), the purported ability of Graciano variety to tolerate relative high UV-B, and/or a combination of them. This may imply that UV-B tolerance is cultivar-dependent, or that UV-B response interacts with other environmental variables not taken into account in these experiments.

## Altitude and Chemical Composition of Grapes and Wines

Most of the wine organoleptic characteristics, namely color, aroma, primary flavors and mouthfeel sensations come from grape biochemical characteristics at harvest (Garrido and Borges, 2013; Wang and Spence, 2018). The chemical components of grapes that are relevant during winemaking are sugars, phenolic compounds, organic acids and volatile organic compounds (Garrido and Borges, 2013; do Nascimento Silva et al., 2015; González-Barreiro et al., 2015). Figure 2C provides an overview on how lower temperature and higher UV-B radiation found in high altitude vineyards affect the concentration of these compounds.

Grape sugar content is directly related to the metabolite synthesis, transport and tissue accumulation (Murcia et al., 2015; Mansour et al., 2022). Additionally, grape sugar measured as °Brix with a refractometer can be indirectly affected by changes in water content and berry size. Low temperatures at high altitudes considerably delay berry maturation, plus lower net photosynthesis in leaves exposed to higher UV-B causes diminished sugar accumulation (Berli et al., 2011). However, that does not necessarily affect the sugar concentration expressed as °Brix, since major evaporative losses are observed, increasing dry matter concentration (Keller, 2010). Moreover, berry size tends to decrease with increasing altitude as a result of higher oxidative damage, which ultimately contributes to an enhanced sugar accumulation per berry (Berli et al., 2011).

According to Robinson et al. (2011), in a study comparing the influence of site, canopy management and yeast strain, the major factor influencing the volatile composition and sensory characteristics of a wine is the vineyard location, which can produce differences in up to 73% of wine volatile organic compounds levels. Aside from site specific characteristics, such as soil composition and terrain aspect, the main environmental variables that affect grape biochemistry are temperature and light exposure (Robinson et al., 2011; Garrido and Borges, 2013; González-Barreiro et al., 2015). As noted before, these vary somewhat predictably with increasing altitude (Körner, 2007), but may vary in a more different way by annual local changes in environmental conditions.

Phenolic compounds are one of the most abundant and important type of secondary metabolites in grape and wine, being mainly constituted of hydroxycinnamic acids and flavonoids (Garrido and Borges, 2013). There are three main groups of flavonoid compounds: proanthocyanidins, anthocyanins and flavonols, and they have important physiological functions during berry development, such as antioxidant activity, UV radiation protection and defense against microbial and fungal infection (Sun et al., 2017). Additionally, anthocyanins mostly contribute to pigmentation in wines, while proanthocyanidins and flavonols are responsible for some major wine sensorial properties such as astringency and turbidity (Blancquaert et al., 2019).

Environmental factors, mainly light and temperature, have been acknowledged to influence phenolics composition of grape and wines (Sun et al., 2017). Studies concerning vineyard altitude have consistently shown a positive correlation between elevation and the production of both anthocyanins and flavonols (Berli et al., 2008; Li et al., 2011; Liang et al., 2014). In a series of growth chamber experiments directed to determine the optimum temperature range for anthocyanin and flavonol accumulation in Merlot grapes, Yan et al. (2020) found that a higher thermal amplitude, as well as lower night temperature resulted in the highest flavonol and anthocyanin levels. This was related to a twofold higher *VviMybA* expression, a central anthocyanin transcription factor, during the cold temperature regime.

Particularly, higher altitude tends to promote cyanidin-type or non-acylated anthocyanins (Xing et al., 2016; Muñoz et al., 2021; Urvieta et al., 2021). Certainly, UV-B has been pointed out to be the main contributor to this effect by direct activation of the flavonoid 3′-hydroxylase (F3′H)-mediated branch of the phenylpropanoid pathway in berry skins, which produces 3′,4′-hydroxylated flavonoids such as quercetin-type flavonols, cyanidin-type anthocyanins, catechin and epicatechin (Berli et al., 2011; Martínez-Lüscher et al., 2014; Xing et al., 2016).

In a UV-B exclusion experiment, Malbec grapevines that were exposed to sunlight UV-B at 1500 m a.s.l. had 17.6% more total polyphenols and 28.5% more total anthocyanins in the berry skins as compared to those of the minus UV-B treatment (Berli et al., 2008). In very similar setup, Tempranillo berries at 371 m a.s.l. only responded slightly to the UV-B exclusion, with a positive correlation only for UV dose with the flavonols quercetin and kaempferol contents (Del-Castillo-Alonso et al., 2016). This may be attributed to the quite low UV-B dose at the altitude of 371 m a.s.l., rather than a speculative assumption that Tempranillo variety is well adapted to UV-B.

Temperature, on the other hand, seems to follow a negative correlation with flavonoid concentration (Pastore et al., 2017). This is likely because of temperatures over 40°C inhibit the phenylalanine ammonia lyase (PAL) and the stilbene synthase, key enzymes of the phenylpropanoid pathway (Ferrandino and Lovisolo, 2014; Pastore et al., 2017). Mori et al. (2007) linked this inhibitory effect of high temperature to both a lower mRNA accumulation of anthocyanin pathway genes for certain varieties, and a selective degradation of the less stable non-methylated anthocyanins. This results in an overall lower anthocyanin content along with a change in hue favored by highly methylated malvidin derivatives. As stated by Drappier et al. (2019), for optimal anthocyanin accumulation bunches need to be exposed to 15°C nights and 25°C daytime temperatures during ripening. Accordingly, Urvieta et al. (2021) found lower anthocyanin accumulation in Malbec grapes and wines cultivated at lower altitude sites of Mendoza (635 m a.s.l), where the number of days with temperatures above 33°C was significantly higher as compared to higher altitude sites (1,500 m a.s.l). Movahed et al. (2016) directly linked this phenomenon to both the inhibition of biosynthesis by transcriptional regulation, and the gene activation of peroxidases during heat stress, which mediate anthocyanin degradation. In a field study with a simulated 2–3°C warmer climate than control, de Rosas et al. (2017) found a significant reduction of total anthocyanins (28–41%) and a higher proportion of acylated anthocyanins in Malbec and Bonarda grape skins. This correlated with lesser expression of regulatory and structural anthocyanin genes. Conversely, cooler temperatures are known to improve color and stability in wines (González-Barreiro et al., 2015). Falcão et al. (2010) associated this phenomenon with the fact that bunches remain attached longer to the vine, thus having an extended veraison to harvest phenophase to accumulate colored compounds in response to light. In addition, a smaller berry caused by high altitude conditions could tilt polyphenolic compounds to a higher concentration when analyzed as per berry basis (Berli et al., 2011). Touriga Nacional and Touriga Francesa varieties cultivated at 300–350 m a.s.l. contained up to 59% more total anthocyanins than those grown at 100–150 m a.s.l., supposedly due to a 5°C mean temperature differential (Mateus et al., 2001a, 2002). The authors argued that anthocyanins, interacting with the also greater amount of the flavanols catechins and procyanidins, improve color stabilization and aging capacity in the wine made from berries grown at the higher altitude (Mateus et al., 2001b). Contrary to most literature, Pajovic et al. (2014) found for Vranac, Kratosija and Cabernet Sauvignon varieties lower total polyphenols and both monomeric and polymeric proanthocyanidins in grapes cultivated at 400 m a.s.l. as compared to those grown at 25 m a.s.l. The authors proposed that these results were attributed to the fact that cultivation was done in a quite cool region, and therefore low temperatures were detrimental for secondary metabolism. The higher polyphenol contents at high altitudes might also be related to activation of ABA dependent abiotic stress responses to cold temperatures, which ultimately lead to accumulation of antioxidants such as glutathione, carotenoids and flavonoids (Ferrandino and Lovisolo, 2014). The positive relationship between altitude-related environmental factors and the antioxidant defense system has been documented for different cultivars and regions (Berli et al., 2015; Coklar et al., 2017), generally pointing to an increase in phenolic compounds with antioxidant activity, but also to a higher activity of antioxidant enzymes as a response to the combined effect of higher UV-B and lower temperature generating reactive oxygen species.

Wine pH determines the equilibrium between different anthocyanin structures. In very acidic conditions, the flavylium cation (red) is the main anthocyanin structure, but it is progressively replaced by the quinoidal base (blue) as the pH increases (Kontoudakis et al., 2011). Therefore, the higher titratable acidity and lower pH found in musts of certain cultivars grown at higher as compared to lower temperatures may help wines retain its red hue. Although the relationship between acidity and temperature seems to be highly cultivar-dependent (Sadras et al., 2013), it has been argued that low temperatures imply less temperature-dependent malic acid breakdown, sufficient to increase overall must acidity (Koundouras et al., 2006).

The volatile organic compounds responsible for wine aromatic quality are also influenced by climatic variables during berry maturation. The levels of monoterpenes in pre-harvest grapes increased by UV-B both *in-vitro* (irradiated bunches) and in field conditions (UV-B exclusion at 1450 m a.s.l.; Gil et al., 2013). These compounds have strong antioxidant properties and are synthesized in grapes as a response to stress-inducing conditions, such as insect herbivory, wounding or high UV-B (Gil et al., 2012, 2014).

In general, high temperatures lead to less intense aromas such as pyrazines or monoterpenes, and might promote undesirable flavors such as o-Aminoacetophenone, a compound whose aroma is often described as varnish of mothball like (De Orduña, 2010). Yue et al. (2015) demonstrated by solid phase microextraction coupled with gas chromatography–mass spectrometry (SPM-GC/MS) that the number of volatile compounds in Cabernet Sauvignon wines increased with altitude (from 2,110 m to 2,778 m a.s.l.). In a similar experiment, Cabernet Sauvignon wines from 1,415 m a.s.l. were strongly associated to bell pepper aroma, and spice aroma to a lesser extent, while wines from 774 m a.s.l. were marked by red fruits, jam and toasted aroma attributes (Falcão et al., 2007). The chemical component responsible for bell pepper aroma, 2-methoxy-3-isobutylpyrazine (MIBP), was positively associated by regression to vineyard altitude and lower seasonal temperature (Falcão et al., 2007). Pyrazine’s accumulation is known to be lower with cooler climates (Romero et al., 2006; Plank et al., 2019), and their degradation has been linked to sunlight exposure (Koch et al., 2012; Sivilotti et al., 2016). Furthermore, after a sensory evaluation of Merlot and Cabernet Sauvignon wines from 2,282, 2,435 and 2,608 m a.s.l., the trained panelists scored higher those wines coming from higher altitudes, highlighting an improvement in characteristics such as taste quality and aroma intensity (Jin et al., 2017).

Martínez-Lüscher et al. (2015) investigated the combined effects of elevated temperature, CO_2_ and UV-B radiation for Tempranillo grape and leaf development. The hastened grape ripening caused by higher carbon fixation due to increased temperature and CO_2_ was attenuated by UV-B radiation (Martínez-Lüscher et al., 2016b). This was attributed to a down-regulation of photosynthesis during the first days of exposure to UV-B before veraison (Martínez-Lüscher et al., 2016b). Concomitantly, the lower anthocyanin to sugar ratio result of higher carbon fixation was alleviated by UV-B radiation directly activating the anthocyanin biosynthesis pathway, thus reversing the uncoupling (Martínez-Lüscher et al., 2016b). Moreover, exposure to higher UV-B radiation elicited enzymatic and non-enzymatic antioxidant responses, contributing to a reduction of oxidative damage induced by higher temperature (Martínez-Lüscher et al., 2015).

Van Leeuwen and Destrac-Irvine (2017) argued that the increase in concentration of skin phenolics, aroma precursors and volatile compounds result of direct radiation exposure could enhance grape quality for red wine production, but at the same time be a drawback in producing white wines. Furthermore, higher levels of radiation before veraison could cause sunburn damage, impairing grape quality for both red and white varieties (Van Leeuwen and Destrac-Irvine, 2017). Consequently, certain canopy management strategies such as leaf pulling should be avoided, favoring interventions that result in shading of grape bunches (Van Leeuwen and Destrac-Irvine, 2017).

Gaiotti et al. (2018) assessed how cooler night temperatures at higher altitudes affect grape ripening and coloration. Low night temperatures of 10–11°C slows down the malic acid degradation, while increasing anthocyanin accumulation when imposed around veraison, resulting in higher anthocyanin content in skins at harvest date (Gaiotti et al., 2018).

## Conclusion

Climate is progressively getting warmer and with more extreme events due to a continued increase in greenhouse gas emissions from anthropogenic activities. Even under very low emissions future scenarios, temperatures are estimated to remain elevated until at least 2,100. This is driving a shift in agricultural land use to accommodate for each crop requirements. Grapevine cultivation for winemaking is very susceptible to climatic variation, given that the terroir effect is paramount in producing specific wine styles. In order to maintain proper growing temperatures, vineyards are currently experiencing an expansion towards higher altitudes, where not only mean temperatures are lower, but there is a higher thermal amplitude and global radiation, particularly in the UV spectrum. These altitude-related climatic factors, when integrated, tend to cause a general delay in the growth cycle, reduced vegetative growth and berry size, along with an enhanced antioxidant system, both through higher accumulation of phenolic compounds and higher activity of antioxidant enzymes. Additionally, under higher altitudes, grapes display higher anthocyanin content and higher acidity, alleviating the effects of CC conditions that cause a premature ripening. Lastly, wines produced from high altitude vineyards tend to have better color, higher acidity and more desirable aroma profiles.

## Author Contributions

LA: conception, data collection, and redaction. FB, AF, RB, and PP: critical revision from draft to version to be published. All authors reviewed and approved the final version of the manuscript.

## Funding

This work has been supported by Grant PICT 2019-1722 from Consejo Nacional de Investigaciones Científicas y Técnicas (CONICET, Argentina) and Secretaría de Ciencia y Técnica de la Universidad Juan A. Maza. LA is a recipient of a CONICET scholarship. FB, AF, RB, and PP are fellows of CONICET.

## Conflict of Interest

The authors declare that the research was conducted in the absence of any commercial or financial relationships that could be construed as a potential conflict of interest.

## Publisher’s Note

All claims expressed in this article are solely those of the authors and do not necessarily represent those of their affiliated organizations, or those of the publisher, the editors and the reviewers. Any product that may be evaluated in this article, or claim that may be made by its manufacturer, is not guaranteed or endorsed by the publisher.
